# Multimode Fabry–Perot Interferometer Probe Based on Vernier Effect for Enhanced Temperature Sensing

**DOI:** 10.3390/s19030453

**Published:** 2019-01-22

**Authors:** André D. Gomes, Martin Becker, Jan Dellith, Mohammad I. Zibaii, Hamid Latifi, Manfred Rothhardt, Hartmut Bartelt, Orlando Frazão

**Affiliations:** 1 Leibniz Institute of Photonic Technology (IPHT), Albert-Einstein-Strasse 9, 07745 Jena, Germany; martin.becker@leibniz-ipht.de (M.B.); jan.dellith@leibniz-ipht.de (J.D.); manfred.rothhardt@leibniz-ipht.de (M.R.); hartmut.bartelt@leibniz-ipht.de (H.B.); 2 INESC TEC and Department of Physics and Astronomy, Faculty of Sciences, University of Porto, Rua do Campo Alegre 687, 4169-007 Porto, Portugal; ofrazao@inesctec.pt; 3 Laser and Plasma Research Institute, Shahid Beheshti, Evan, Tehran 1983969411, Iran; m_zibaye@sbu.ac.ir (M.I.Z.); latifi@sbu.ac.ir (H.L.)

**Keywords:** optical fiber sensor, Fabry–Perot interferometer, temperature sensing, focused ion beam, Vernier effect

## Abstract

New miniaturized sensors for biological and medical applications must be adapted to the measuring environments and they should provide a high measurement resolution to sense small changes. The Vernier effect is an effective way of magnifying the sensitivity of a device, allowing for higher resolution sensing. We applied this concept to the development of a small-size optical fiber Fabry–Perot interferometer probe that presents more than 60-fold higher sensitivity to temperature than the normal Fabry–Perot interferometer without the Vernier effect. This enables the sensor to reach higher temperature resolutions. The silica Fabry–Perot interferometer is created by focused ion beam milling of the end of a tapered multimode fiber. Multiple Fabry–Perot interferometers with shifted frequencies are generated in the cavity due to the presence of multiple modes. The reflection spectrum shows two main components in the Fast Fourier transform that give rise to the Vernier effect. The superposition of these components presents an enhancement of sensitivity to temperature. The same effect is also obtained by monitoring the reflection spectrum node without any filtering. A temperature sensitivity of −654 pm/°C was obtained between 30 °C and 120 °C, with an experimental resolution of 0.14 °C. Stability measurements are also reported.

## 1. Introduction

Biological and medical applications require minimally invasive sensors, especially for in-vivo operation. Therefore, the current tendency is towards developing miniaturized sensors that are capable of measuring physical, chemical, and biochemical parameters. For this purpose, Fabry–Perot interferometers (FPIs) are a type of sensing structure that has been explored due to the ability of producing sensing probes that can be optically interrogated in a reflection configuration. 

Traditional FPIs involve the creation of a diaphragm at the end of an optical fiber [1]. New FPI configurations using different types of fibers, or even ablation techniques to structure fiber tips, allowed for measuring different sensing parameters. Hollow-core silica tube FPIs [2], silicon-core FPIs [3], photonic crystal fiber FPIs [4], and femtosecond laser-milled FPIs [5] are some examples of structures proposed. Focused ion beam-milled FPI cavities in fiber tips have also been explored, especially regarding the advantage of creating small sensing structures in thin fiber tips, which can be smaller than a single cell. For example, air and silica FPIs in fiber tips were demonstrated in 2016 [6]. Ultra-short FPI cavities milled in microfibers were also proposed as miniaturized sensing devices [7]. 

In terms of temperature sensing, conventional silica FPIs are limited by the thermo-optic coefficient of silica and to a lesser degree by the thermal expansion coefficient. Typical temperature sensitivity values for these structures range from 10 pm/°C to around 20 pm/°C [2,6,8,9]. To overcome this problem, polymers started to be implemented. Polymer FPIs can attain temperature sensitivities that are one order of magnitude higher due to their high thermal expansion coefficient [10,11].

However, their use is also limited to temperatures below the polymer melting point. Apart from polymers, other possibilities to break this limit include creating sensing structures that present other effects beyond the normal FPI, such as multimode interference [12], or show a non-linear response behavior [10,12].

Recently, the Vernier effect has increasingly been used in optical fiber sensing due to its ability to enhance the measurement sensitivity [13,14,15,16,17]. With this, interferometric sensing structures can achieve higher spectral shift, allowing for higher resolution measurements [18]. The Vernier effect occurs in the presence of two interferometers with slightly shifted frequencies. The superposition of the two spectra originates a beat between the two frequencies, producing a large envelope with interesting properties. For instance, the spectral shift that is caused by the measurands is magnified in the envelope when compared with the normal shift of the single interferometers [18].

In this work, we apply the concept of the Vernier effect to produce a focused ion beam-milled silica Fabry–Perot interferometer probe that is capable of achieving an enhanced spectral shift to temperature compared to the conventional silica cavity in a fiber tip. The silica Fabry–Perot interferometer is created at the end of a tapered multimode fiber tip. Multiple individual interferometer effects with shifted frequencies are created, generating beats between them. The envelope presents a node that shows a magnified wavelength shift to temperature due to the Vernier effect.

## 2. The Optical Fiber Probe

### 2.1. Fabrication Process

The fiber probe was fabricated using a step-index multimode optical fiber (MMF) (FG050LGA, Thorlabs GmbH, Munich, Germany) with an inner core diameter of 50 µm and a standard outer diameter. The MMF was tapered down using a CO_2_ laser, which created a sharp tip that is suitable to be post-processed. Afterwards, the sensing structure was created at the MMF tip by focused ion beam milling. 

To use such technique, it is necessary to deposit a thin layer of a conductive material (carbon) on the fiber in order to suppress the surface charging effects. Charge accumulation can deviate the ion beam, originating unwanted milled regions and inaccurate milling geometries by drift effects. The fiber was mounted and fixed in an aluminum holder with a droplet of carbon glue (DOTITE XC-12, Fujikura Kasei Co., Ltd., Tokyo, Japan). The whole set was then carbon-coated by means of a LEICA EM ACE600. The sample was placed at a working distance of 50 mm, with a 5° stage tilt. Nine pulses were applied with a chamber pressure of 6 × 10^−5^ mbar to deposit a nearly 6 nm-thick carbon film on the sample.

**Figure 1:**
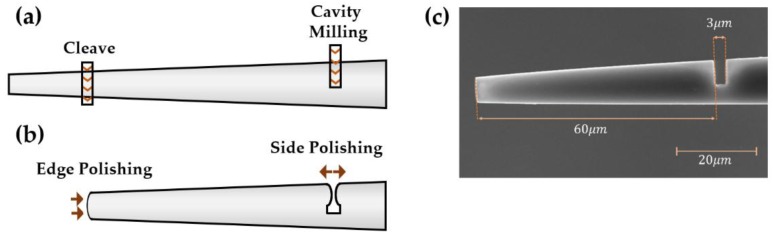
The sensing structure is fabricated by focused ion beam milling. (**a**) Fiber tip cutting and milling of a small air cavity. (**b**) Edge polishing and cavity side polishing. (**c**) Scanning electron microscope image of the final structure.

A Tescan (Lyra XMU) focused ion beam—scanning electron microscope (FIB-SEM)—was used to create a silica cavity in the MMF tip. The fabrication process is illustrated in Figure 1. First, the fiber end was cleaved with an ion current of nearly 1 nA. A 2 µm-wide, 7 µm-high air cavity was milled 60 µm away from the cleaved edge. At the position of the air cavity, the fiber has a diameter of 11.6 µm. The fiber edge was polished using the same current in order to produce a smooth surface that will act as a mirror through Fresnel reflection. During the milling of the cavity, some of the removed material is redeposited in the side walls, creating an irregular surface. Therefore, it is essential to polish the cavity walls to create smooth surfaces, preventing light from being scattered to the outside. The side polishing was performed with a current of 800 pA. 

The sensor was disassembled from the aluminum holder by acetone to remove the carbon glue. The whole structure was placed in an ultrasonic bath with acetone for 10 minutes in order to clean the sensing head and remove carbon glue residues. 

**Figure 2:**
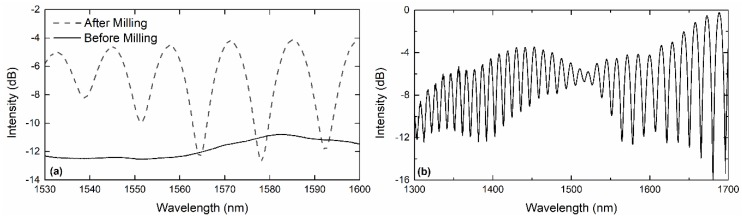
(**a**) Reflection spectrum, before and after milling the cavity. After milling, the reflection spectrum presents an interferometric behavior. (**b**) Reflection spectrum in a broader wavelength range.

Figure 1 also shows a scanning electron microscope image of the final MMF probe. The fiber probe is comprised of a 2.7 µm-wide air cavity and a 60.2 µm-wide silica cavity being located between the air cavity and the polished fiber end. Note that the final height of the air cavity is 6.1 µm rather than the predicted 7 µm due to material redeposition during the milling process. Figure 2a shows the reflection spectrum of the structure before and after milling. In reflection, the silica and air cavities work as Fabry–Perot interferometers, and so the reflection spectrum presents an interferometric behavior. 

### 2.2. Working Principle

As explained before, both the air and silica cavities act as Fabry–Perot interferometers, where light is partially reflected at each air-silica interface through Fresnel reflections. However, since the reflection at each interface is very small (less than 3.5%), the response can be approximated as a two-wave interferometer. The distance between two interference minima, commonly known as the free spectral range (FSR), is given by [19]:

(1) $$\mathrm{FSR}=\frac{\lambda_{1}\lambda_{2}}{2n_{eff}L},$$

where $\lambda_{1}$ and $\lambda_{2}$ are the positions of the minima, $n_{eff}$ is the effective refractive index, and *L* is the cavity length. The FSR corresponding to the normal Fabry–Perot interferometer given by the silica cavity is around 12.9 nm. However, due to the presence of multiple modes, the silica cavity produces the effect of several Fabry–Perot interferometers, one for each mode, with slightly different frequencies. The superposition of all interferometers results in different beatings, producing a complex envelope that shows a node, as seen in Figure 2b. 

As seen before, the Vernier effect is generated in the presence of two interferometers with shifted frequencies, producing a large envelope that shows a magnification of the spectrum shift. However, the Vernier effect is also presented in more complex structures, such as multi-beam interferometers, where the interference between a fundamental mode and several higher order modes is observed [17], such as the structure proposed in this work.

**Figure 3:**
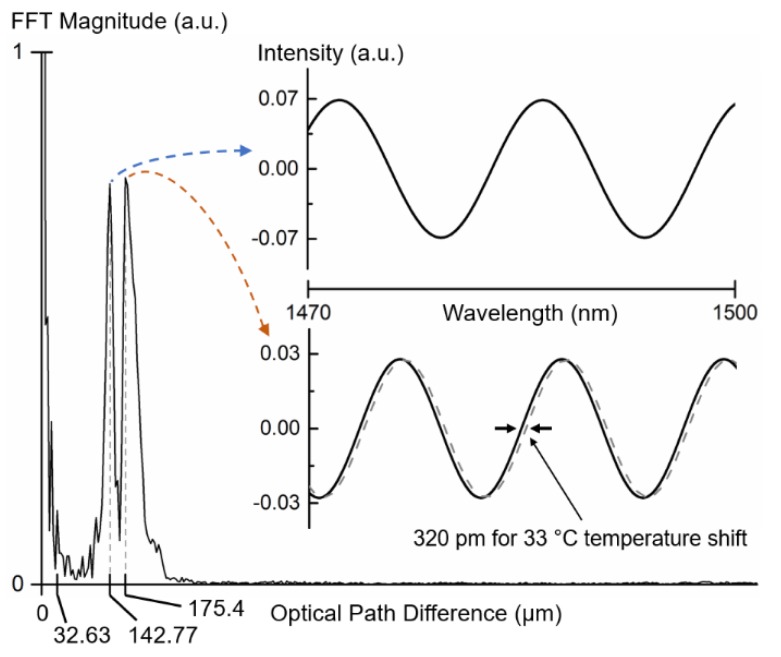
Fast Fourier transform of the reflection spectrum. Inset: Predominant components of the reflection spectrum, corresponding to the two peaks presented in the fast Fourier transform (FFT). The wavelength shift with temperature for corresponding to the normal Fabry–Perot interferometer is 9.7 pm/°C.

In order to understand the origin of this node in the reflection spectrum, a fast Fourier transform (FFT) was performed. When considering that the range of wavelengths used in the measurements is very broad, to perform a fast Fourier transform, one needs to make sure that the spacing between the interference fringes (FSR) is the same in all regions of the spectrum, eliminating its wavelength dependence. To do so, the wavelength data were converted into optical frequencies (*v* = *c*/*f*), where *c* is the speed of light in vacuum. Between two interference minima, the phase change is equal to 2*π*, which in terms of optical frequencies can be translated to:

(2) $$\Delta \varphi =2\pi =\frac{2\pi }{\lambda_{1}}n_{eff}2L-\frac{2\pi }{\lambda_{2}}n_{eff}2L=\frac{2\pi \nu_{1}}{c}n_{eff}2L-\frac{2\pi \nu_{2}}{c}n_{eff}2L,$$

where *n_eff_* is assumed the same for both wavelengths. In this new scale, the free spectral range in frequency (*FSR_v_* = *v*_1_ − *v*_2_) is now given by [7]:

(3) $$FSR_{\nu }=\frac{c}{2n_{eff}L}↔OPD=2n_{eff}L=\frac{c}{FSR_{\nu }},$$

where *OPD* is the optical path difference. The FFT of the reflection spectrum in optical frequencies can now be represented in terms of optical path difference, since the FFT X-axis represents an inverse unit of frequency. The result is shown in Figure 3. 

Note that three distinct physical cavities are presented in the structure: the air cavity (*OPD* = 2 × 3 μm), the silica cavity (*OPD* = 2 × 1.444 × 60 μm), and the air cavity together with the silica cavity (*OPD* = 2 × 3 μm + 2 × 1.444 × 60 μm). The optical path difference of the air cavity is very small (6 μm), which produces an interference signal with a long FSR, enough not to have a major impact in the reflection spectrum. This peak is masked by the peak at zero *OPD*. The optical path difference of the silica cavity corresponds to the FFT peak at 175.4 µm. It is clearly visible that this peak extends to slightly higher optical path differences, which corresponds to the combination of the air plus silica cavities, as discussed before. For the analysis performed next, the effect of the air cavity in the silica cavity OPD can be neglected, since the air gap is very small [1].

The reflection spectrum exhibits two major components (two FFT peaks with high magnitude). Both components arise from the silica cavity and from the fact that in the structure multiple modes can propagate, as discussed previously. The effective refractive index difference between the two major components is 0.26. In reality, the reflection spectrum does not resemble the beating between just two frequencies but rather consists of different interferometers of shifted frequencies, originating multiple beats between them. However, note that many of the envelopes that are produced in these multiple interferences have longer FSR, and so they are not clearly visible within the wavelength window used for the measurements. Therefore, the FFT only shows the contribution of the components that fit to the wavelength range used. The superposition of the two main components in the FFT causes the reflection spectrum node, as previously seen.

To prove the existence of the Vernier effect in the structure, let us just consider the two main frequencies of the spectrum. An FFT bandpass filter was applied to the measured FPI’s reflection spectrum (Figure 2b) to isolate the two main components with OPDs equal to 175.4 µm and 142.77 µm, respectively. The result is depicted in the inset of Figure 3.

**Figure 4:**
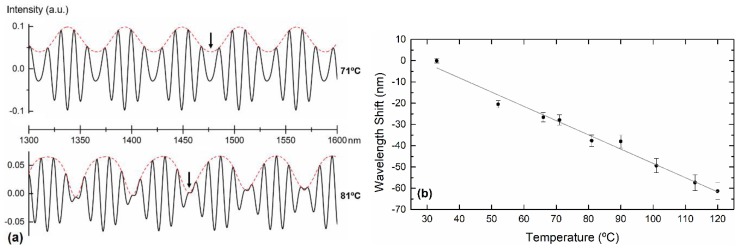
(**a**) Superposition of the two main components filtered from reflection spectrum, at different temperatures. (**b**) Wavelength shift of the envelope minimum as a function of temperature. The envelope shows a temperature sensitivity of (−670 ± 33) pm/°C.

The superposition between the two main frequencies filtered from the reflection spectrum generates an envelope, due to the Vernier effect, that shows a wavelength shift with temperature, as presented in Figure 4. The envelope originates from the beating between the two main frequencies and presents a frequency that is equal to the difference between the frequencies of both components. Such frequency can also be seen in the FFT of the reflection spectrum (*OPD* = 32.63 μm). The temperature sensitivity of the envelope minimum is −670 pm/°C between 30 °C and 120 °C, much higher than the normal wavelength shift of the normal Fabry–Perot interferometer (9.7 pm/°C at 1485 nm, as shown in Figure 4).

**Figure 5:**
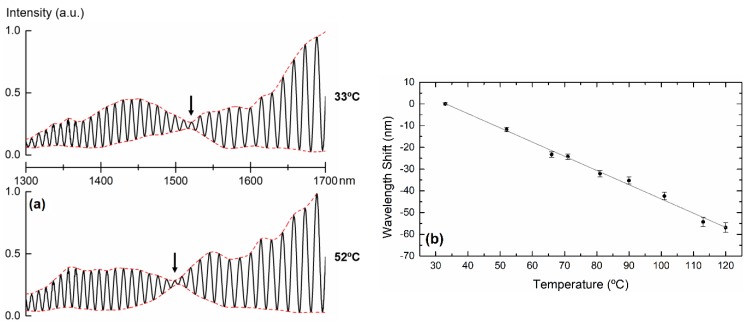
(**a**) Reflection spectrum at two distinct temperatures. The node, marked with an arrow, changes with temperature. (**b**) Wavelength shift of the reflection spectrum node as a function of temperature. The slope corresponds to a temperature sensitivity of (−654 ± 19) pm/°C.

Instead of relying on filtering processes, one can simply obtain the same response by monitoring the envelope node in the reflection spectrum, as observed in Figure 5.

## 3. Temperature Characterization

The reflected interferometric signal was measured by means of a supercontinuum optical source (Fianium, WL-SC-400-2, NKT Photonics GmbH, Cologne, Germany), an optical circulator, and an optical spectrum analyzer (OSA, Yokogawa Electric Corporation, Tokyo, Japan). For practical applications, alternative configurations involving less expensive commercial devices, for example, with a tunable laser can be implemented. The signal was previously normalized, with the reflected signal from a cleaved single mode fiber in air (around 3.5% Fresnel reflection) being taken as a reference. The fiber probe was submitted to different temperatures, ranging from 30 °C to 120 °C, in a tubular oven (Ströhlein Instruments). The position of the node was tracked, without the use of any filters, as a function of temperature by following the average of the minimum of the upper envelope and the maximum of the lower envelope. The wavelength shift of the node is depicted in Figure 5. A linear fit was applied to the experimental data, obtaining a temperature sensitivity of (−654 ± 19) pm/°C.

**Figure 6:**
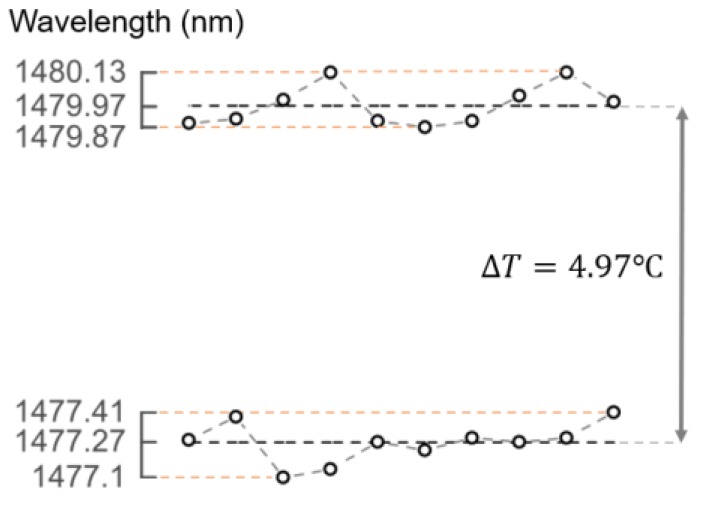
Stability measurements: 10 measurements at two distinct temperatures, 89.54 °C and 94.51 °C.

To evaluate the stability of the sensing structure and determine the detection accuracy, 10 consecutive measurements were performed at two different temperatures, 89.54 °C and 94.51 °C. To ensure a good thermal stability, the sensor was placed in an aluminum box inside a Carbolite oven with high volumetric capacity. The aluminum box partially attenuates the thermal fluctuations of the oven caused by its PID control. A PT100 thermometer was also placed inside the aluminum box together with the MMF probe to help in monitoring thermal stability. To make sure that a good thermal equilibrium was achieved, the oven was allowed to stabilize for 4 h at each temperature before the measurements were performed. The stability results are shown in Figure 6. The maximum standard deviation achieved was 96.98 pm, which corresponds to a detection accuracy of 0.14 °C. The value obtained corresponds to half of the OSA resolution used for the measurements (200 pm). Therefore, the resolution is limited by the resolution of the interrogation system. The theoretical detection limit for the fiber probe, while considering an interrogation system with a resolution of 10 pm, would be 0.015 °C. Such wavelength resolution can be achieved by modern high resolution OSA systems.

Table 1 compares the sensitivity values and resolution for different Fabry–Perot configurations. The proposed fiber probe presents higher temperature sensitivity than many reported Fabry–Perot configurations, especially when considering that it is a silica sensor.

## 4. Conclusions

We developed a small-size fiber probe to generate the Vernier effect by structuring a Fabry–Perot interferometer with focused ion beam milling at the end of a tapered multimode tip. The reflection spectrum shows a node with a magnified spectral shift to temperature. The two main components of the reflection spectrum can be filtered, and their superposition generates a large beating envelope that presents the Vernier effect. The same response can be obtained at the reflection spectrum node, despite presenting a more complex Vernier effect due to the presence of the multiple Fabry–Perot interferometers with slightly different frequencies. At the reflection spectrum node, a temperature sensitivity of (−654 ± 19) pm/°C was achieved between 30 °C and 120 °C. This value is more than 60-fold the sensitivity of a conventional silica Fabry–Perot interferometer (9.7 pm/°C). Stability measurements were also carried out, obtaining a maximum standard deviation of 96.98 pm, corresponding to a detection accuracy of 0.14 °C. This value is however limited by the resolution of the interrogation system. The theoretical resolution of the sensor is 0.015 °C for an interrogation system with a resolution of 10 pm. The fiber probe is suitable for measuring temperature variations in small scale environments, which can be useful, especially in biological applications. Moreover, the temperature of operation of the structure includes typical temperature ranges that are used in biological applications (30 °C+). The next step for future studies is to analyze the response of the sensing structure if it is immersed in liquids with different refractive indices.

## Figures and Tables

**Table 1:** Table of comparison between different configurations. NL stands for non-linear response.

	Sensitivity (pm/°C)	Temp. Range (°C)	Resolution (°C)
FIB-milled FP modal interferometer (2010) [9]	20	19–520	-
Polyvinyl alcohol FPI (2012) [10]	173.5 (NL)	>80	-
SMF + etched P-doped fiber FPI (2014) [20]	11.5–15.5	100–550	-
Silicon FPI (2015) [3]	82	10–100	0.3
Silicon FPI (2015) [21]	84.6	20–100	6 × 10^−4^
Hollow-core FPI with Vernier effect (2015) [17]	816.65	20–90	-
Hollow-core FPI with Vernier effect (2015) [17]	1019	250–300	-
FIB-milled silica FPI in fiber taper (2016) [6]	15.8	40–140	-
Double polymer-capped FPI (2017) [11]	689.68	20–75	-
MMF tip FPI + UV adhesive (2017) [12]	213 (NL)	55–85	-
Cascaded FPI with Vernier effect (2018) [13]	−97	30–60	-
Cascaded FPI with polymer, with Vernier effect (2018) [14]	67,350	20–25	-
**This work**	**−654**	**30–120**	**0.14**
